# DNA-Interacting Characteristics of the Archaeal Rudiviral Protein SIRV2_Gp1

**DOI:** 10.3390/v9070190

**Published:** 2017-07-18

**Authors:** Eveline Peeters, Maarten Boon, Clare Rollie, Ronnie G. Willaert, Marleen Voet, Malcolm F. White, David Prangishvili, Rob Lavigne, Tessa E. F. Quax

**Affiliations:** 1 Research Group of Microbiology, Department of Bio-Engineering Sciences, Vrije Universiteit Brussel, Pleinlaan 2, B-1050 Brussels, Belgium; Eveline.Peeters@vub.be; 2 Laboratory of Gene Technology, Department of Biosystems, KU Leuven, Kasteelpark Arenberg 21 box 2462, Heverlee, 3001 Leuven, Belgium; maarten.boon@kuleuven.be (M.B.); marleen.voet@kuleuven.be (M.V.); rob.lavigne@kuleuven.be (R.L.); 3 Biomedical Sciences Research Complex, University of St Andrews, Fife, North Haugh, St. Andrews KY16 9AJ, UK; cr267@st-andrews.ac.uk (C.R.); mfw2@st-andrews.ac.uk (M.F.W.); 4 Alliance Research Group VUB-UGhent NanoMicrobiology, IJRG VUB-EPFL, BioNanotechnology & NanoMedicine, Research Group Structural Biology Brussels, Department of Bio-Engineering Sciences, Vrije Universiteit Brussel, Pleinlaan 2, B-1050 Brussels, Belgium; Ronnie.Willaert@vub.ac.be; 5 Department of Microbiology, Institut Pasteur, 75015 Paris, France; david.prangishvili@pasteur.fr

**Keywords:** archaea, archaeal virus, *Rudiviridae*, SIRV2, *Sulfolobus*, DNA binding, helix-turn-helix domain

## Abstract

Whereas the infection cycles of many bacterial and eukaryotic viruses have been characterized in detail, those of archaeal viruses remain largely unexplored. Recently, studies on a few model archaeal viruses such as SIRV2 (*Sulfolobus islandicus* rod-shaped virus) have revealed an unusual lysis mechanism that involves the formation of pyramidal egress structures on the host cell surface. To expand understanding of the infection cycle of SIRV2, we aimed to functionally characterize *gp1*, which is a SIRV2 gene with unknown function. The SIRV2_Gp1 protein is highly expressed during early stages of infection and it is the only protein that is encoded twice on the viral genome. It harbours a helix-turn-helix motif and was therefore hypothesized to bind DNA. The DNA-binding behavior of SIRV2_Gp1 was characterized with electrophoretic mobility shift assays and atomic force microscopy. We provide evidence that the protein interacts with DNA and that it forms large aggregates, thereby causing extreme condensation of the DNA. Furthermore, the N-terminal domain of the protein mediates toxicity to the viral host *Sulfolobus*. Our findings may lead to biotechnological applications, such as the development of a toxic peptide for the containment of pathogenic bacteria, and add to our understanding of the Rudiviral infection cycle.

## 1. Introduction

Archaeal viruses display a high morphological and genetic diversity. They represent a separate group, distinct from bacterial and eukaryotic viruses [1]. Amongst the unique morphologies described exclusively for archaeal viruses are spindle-, egg-, spiral- and bottle-shaped virions. Viruses infecting archaea represent the most recently discovered viruses and the limited number of viruses isolated to date is expected to represent only a small fraction of a diverse unexplored world of novel viral families [2].

The large majority of archaeal viruses have double-stranded (ds) DNA genomes, which can be either circular or linear. The sequences of most genes encoded by these genomes yield no hits in extant databases and their functions remain largely unknown [1,2,3]. Studies on the infectious biology of archaeal viruses are hampered by this low number of functionally characterized viral genes. In addition, the infection cycles of archaeal viruses are mostly unexplored. However, in recent years considerable efforts have been made to unravel the molecular mechanisms underlying infection by archaeal viruses and some have emerged as models for the study of virus–host interactions. An example of such a model is the rudivirus *Sulfolobus islandicus* rod-shaped virus 2 (SIRV2). Characterization of its infection cycle revealed unexpected aspects of its structural organization, and of its entry, replication and egress mechanisms [4,5,6,7]. SIRV2 replicates fast, has a clear and dramatic effect on the host upon infection, and is therefore an appealing model to study crenarchaeal viruses.

The linear dsDNA genome of SIRV2 (35 kb) carries inverted terminal repeats (ITR) and encodes 54 open reading frames (ORFs) [8]. SIRV2 infects the thermoacidophilic archaeon *S. islandicus* LAL14/1, which was isolated from solfatares in Iceland and grows optimally at 78 °C and a pH of 3 [9]. It has stiff rod-shaped virions of about 900 nm in length and 23 nm in diameter [9]. The virions consist of multiple copies of the major capsid protein Gp26 enwrapping the linear dsDNA genome. Interestingly, this genome is organized as A-form DNA inside the viral particle, probably to protect the DNA against the high temperature and low pH of the natural environment of *S. islandicus* [10]. The proteins Gp33 and Gp39 are also part of the SIRV2 virions, although in minor amounts [11]. At each end of the non-enveloped virions three tail fibers are displayed, which consist of multiple copies of the protein Gp38 and are important for virion attachment to the host cell during the entry process [7]. The tail fibers bind specifically to pili-like structures of the host and virions travel along them to the cell surface, where they deliver the DNA into the host cytoplasm by an unknown mechanism [7]. The host genome is then rapidly eliminated and the cell is transformed into an efficient virion-producing factory. SIRV1 is another member of the *Rudiviridae* that is closely related to SIRV2. It was isolated in Iceland at a separate location from SIRV2, and infects *S. islandicus* KVEM10H3. It has a similar genome organization and morphology as SIRV2 [9]. The main difference between SIRV1 and SIRV2 is that SIRV1 encodes nine fewer genes, and that it has an unusual genome instability, which is illustrated by the high number of available genetic variants [8,12]. Therefore, the more stable SIRV2 is more amenable to virus–host interaction studies.

As a first step during archaeal viral infection, the viral genomes are replicated. The genome organization of *Rudiviridae* with their ITRs is reminiscent of that of large cytoplasmic DNA viruses, such as the *Poxviridae* [13]. However, the rudiviruses replicate by a novel mechanism involving a Rep-like protein, Gp16 [6]. Gp17 and Gp18 were also suggested to play roles in replication [14]. During replication, head-to-head and tail-to-tail replicative intermediates are formed, which can be resolved by the virus-encoded Holliday junction resolvase Gp35 [6,15]. After the SIRV genome has been replicated, new linear virions are formed in the cytoplasm of the host cell, by the packaging of the DNA genome with the coat protein Gp26. Simultaneously, preparations are made for virion release. Multiple heptagonal pyramidal-shaped structures are formed on the cell surface [4]. These virus-associated pyramids (VAPs) consist of multiple copies of the virus-encoded membrane protein forming Virus-Associated Pyramids (PVAP) (Gp49) and open outwards creating large apertures (~200 nm) through which the virions can egress [5,16,17]. This unique virus egress mechanism was demonstrated to exist only in a small set of crenarchaeal viruses; i.e., SIRV2 and STIV1 (*Sulfolobus* turreted icosahedral virus) [16,17].

In contrast to most archaeal viruses, quite a number of genes of SIRV2 already have predicted or assigned functions [3]. Still, the functions of about half of all SIRV2 genes are unknown and await functional characterization to obtain further insights into the SIRV infection cycle. One of these uncharacterized proteins is Gp1, named SIRV2_Gp1 throughout this paper to discriminate from its SIRV1 homolog (SIRV1_Gp1). Previously, this protein was also referred to as ORF83a/ORF83b depending on the genomic location of its encoding gene [18]. SIRV2_Gp1 (and SIRV1_Gp1) is encoded twice in the viral genome: both genes have identical DNA sequences and are located at each genome terminus [8,18]. Transcriptomic analysis of the SIRV2 infectious cycle showed that both gene copies are transcribed at very high levels during the very first stages of infection and that their expression levels remain high throughout the infection cycle [18,19].

Gene duplication and high expression levels suggest an important function of SIRV2_Gp1 with regards to the infection process. However, its function remains elusive. Given that it is a small 8-kDa protein almost entirely characterized by a helix-turn-helix (HTH) motif, typical of DNA-binding proteins, we aimed to functionally characterize this protein by studying its putative ability to interact with DNA, using electrophoretic mobility shift assays (EMSAs) and atomic force microscopy (AFM). These investigations showed that SIRV2_Gp1 is capable of binding and condensing dsDNA. Furthermore, by using a *Sulfolobus acidocaldarius* expression system we provided proof that SIRV2_Gp1 is a highly toxic protein although the HTH motif does not seem to contribute to the observed DNA-binding and toxicity characteristics of the protein.

## 2. Materials and Methods

### 2.1. Protein Purification

The *SIRV2_gp1* open reading frame and its truncated variant (*SIRV2_gp1 ∆HTH*) were amplified with primers 1 and 2 and 1 and 22, respectively (Table S1) from Integrated DNA Technologies (IDT, Coralville, IA, USA) and were cloned with C-terminal His-tag in pEXP5-CT/TOPO. The plasmids encoding SIRV1_Gp1 and SIRV1_Gp ∆HTH were transformed into *Escherichia coli* Rosetta™ (DE3)pLysS Competent Cells (Novagen, Madison, WI USA) and BL21 (DE3) pLysS chemically competent cells, respectively. Cells were grown in LB (Luria–Bertani) medium supplemented with 50 μg/mL ampicillin and grown to an optical density of 600 nm (OD_600_) of ~0.4–0.8 at 37 °C. Recombinant protein expression was then induced by the addition of 1 mM isopropyl-β-d-thiogalactopyranoside (IPTG) and cells were grown for 3 more hours at 37 °C. Cells were pelleted and resuspended in lysis buffer (50 mM Tris pH8 500 mM NaCl, 30 mM imidazole, 1 mg/mL lysozyme, protease inhibitor (Roche Applied Science, Basel, Switzerland). Cells were lysed by sonication, the lysate was cleared by ultracentrifugation and the supernatant was filtered through a 0.22 μm syringe filter and loaded on to a 1 mL Protino^®^ Ni-NTAcolumn (Machery-Nagel, Bethlehem, PA, USA) equilibrated in buffer A (50 mM Tris pH 8500 mM NaCl, 30 mM imidazole). SIRV2_Gp1 was eluted with a linear gradient from 30 to 500 mM imidazole. Peak fractions were analyzed by sodium dodecyl sulfate polyacrylamide gel electrophoresis (SDS-PAGE) and the fractions containing the highest amounts of protein were pooled, filtered on a 0.22 μm syringe filter and directly loaded on a HiLoad 16/600 SuperDex 75 pg column (GE Healthcare, Little Chalfont, UK), without prior concentration. The protein was run on the gelfiltration column in buffer C (20 mM MES pH 6.5, 300 mM NaCl, 1 mM DTT, 1 mM EDTA). Purified and concentrated protein samples were flash frozen and stored at −80 °C. The SIRV2_gp1 ∆HTH truncation mutant protein was recombinantly purified following a similar procedure as for the full-length protein with the following change: lysis buffer and Buffer A did not contain imidazole. The *SIRV1_gp1* gene was cloned and the corresponding protein was expressed and purified with immobilized metal affinity chromatography (Ni-IMAC) and gel filtration chromatography as described by Oke et al. [20]. The crystallization and structure solution of SIRV1_Gp1 have been previously described [20], and the coordinates are available from the Protein Data Bank (PDB) (identifier [ID] 2X48).

### 2.2. Electrophoretic Mobility Shift Assays

Different 5′ fluorescein amidite (6-FAM) labeled random 30 bp oligonucleotides were ordered from IDT. Oligos 3–4 (for dsDNA), 5 (for hairpin DNA) and 7–10 (for Holliday junctions) (see Table S1) were annealed by heating with an excess of unlabeled strands at 90 °C for 2 min and then slowly cooling to room temperature overnight in a heating block. In case of single-stranded (ss) DNA, no prior heating occurred and oligo 3 or 4 were used alone. The assembled substrates were purified by native polyacrylamide (12%) gel electrophoresis with 1× Tris-borate-EDTA (TBE) buffer, followed by band excision, gel extraction and ethanol precipitation before being resuspended in water to a concentration of 1 μM for use in assays. The final concentration in assays was 100 nM. Serial dilutions of purified protein and labeled oligonucleotides were mixed in reaction buffer (50 mM Tris pH 7.5, 5 mM EDTA, 1 mM DTT, 100 μg/mL bovine serum albumin (BSA). After a 20 min incubation at room temperature, samples were mixed in a 2:1 ratio with ficoll, loaded on 8% Tris-Borate-EDTA (TBE) gel and electrophoresed at 180 V during 1 to 2 h. After electrophoresis, the gels were scanned using a Fujifilm FLA-5000 imager at a wavelength of 473 nm.

EMSAs with specific DNA fragments were performed as described previously [21]. Briefly, different concentrations of SIRV2_Gp1 or SIRV2_Gp1 ∆HTH protein were mixed with 5′-end ^32^P-labeled probes in presence of an excess of unlabeled salmon sperm DNA (25 ng/μL) in reaction buffer (20 mM Tris pH 8.0, 0.4 mM EDTA, 1 mM MgCl_2_, 0.1 mM DTT, 12.5% glycerol, 50 mM NaCl) and incubated for 25 min at 37 °C prior to analysis by native acryalamide gel electrophoresis. The labeled probes are a 236 bp fragment corresponding to the region upstream of the SIRV2_GP1-encoding ORF (prepared with primers ep399 and ep400, Table S1) and a 173 bp unspecific promoter fragment of *S. acidocaldarius* (prepared with primers ep092 and ep093, Table S1) for SIRV2_Gp1 binding and a 102 bp unspecific fragment of *S. acidocaldarius* (prepared with primers LL139 and LL140, Table S1) for SIRV2_Gp1 ∆HTH binding. Bands were visualized by autoradiography.

EMSAs with plasmid DNA were performed by mixing 100 ng pUC19 DNA (New England Biolabs, Ipswich, MA, USA) with different concentrations of protein in reaction buffer 1 (50 mM Tris pH 7.5, 5 mM EDTA, 1 mM 1,4-Dithiothreitol (DTT), 100 μg/mL BSA) or 2 (20 mM Tris, pH 8.0, 1 mM MgCl, 50 mM NaCl, 0.4 mM EDTA, 0.1 mM DTT, 12.5% glycerol), which gave the same results. After an incubation of 20 min at room temperature, samples were mixed in a 1:5 ratio with 6× DNA loading dye (Thermo Scientific, Waltham, MA, USA) and loaded on an ethidium bromide gel, which was run for 30 min at 100 V after which bands were visualized with an ultraviolet (UV) scanner.

### 2.3. Cleavage Assays

5′-FAM labeled oligonucleotides (see above) and 5 µM of protein were mixed in reaction buffer (20 mM Tris pH 7.5, 10 mM NaCl, 1 mM DTT, 10 mM MgCl) and incubated during 30 min at 50 °C. One unit of Proteinase K was added, samples were incubated at 37 °C and after 30 min, formamide was added 1:2 to the reaction mixture. Samples were loaded on a 20% Urea TBE gel and run at 22 W at 45 °C for 2–3 h.

### 2.4. Atomic Force Microscopy

For AFM imaging, protein-DNA binding mixtures containing 50 nM pUC18 plasmid DNA and 15 nM-30 nM SIRV2_Gp1 protein were prepared in adsorption buffer (40 mM HEPES pH 6.9, 10 mM NiCl_2_) and deposited on freshly cleaved mica. After 5 min incubation, the mica surface was rinsed with deionized ultrapure water and blown dry with a gentle stream of nitrogen. Images were collected with a MultiMode (NanoScope IIIa) AFM (Bruker, Billerica, MA, USA) operated in tapping mode in air using RTESP (Bruker) AFM tips (cantilever length of 115–135 μm, width of 30–40 µm, a nominal spring constant of 20–80 N/m, and resonance frequencies in the range from 264 to 284 kHz). NanoScope Analysis v1.5 software (Bruker) was used to flatten the images, perform cross-section analyses of the complexes, and to make three-dimensional (3D) surface plots of selected complexes with a pitch of 3°.

### 2.5. Toxicity Assay

The *SIRV1_gp1* and *SIRV2_gp1* genes and two truncation mutants of the *SIRV2_gp1* gene lacking 84 bp on the 5′ end (SIRV2_Gp1 ∆N-term) or 117 bp on the 3′ end (SIRV2_Gp1 ∆HTH), were amplified from viral genomic DNA with primers 16 + 17, 1 + 2, 18 + 19 and 20 + 21 respectively (Table S1). The genes were cloned in a pENTR™/SD/D-TOPO^®^ vector according to manufacturer’s protocol and transformed to One Shot^®^ TOP10 Chemically Competent *E. coli* (Thermo Scientific). Next the genes were introduced via Gateway^®^ (Thermo Scientific) cloning in the maltose inducible expression plasmid for *S. acidocaldarius*, pSVA1551 [22]. pSVA1551 harbors the *pyrEF*-encoded proteins, which allow for selection on uracil-free medium when expressed in *S. acidocaldarius* MW001 (Δ*pyrEF*). Plasmids were methylated in *E. coli* ER1828 and 150 ng was transformed to the *S. acidocaldarius* MW001 via electroporation as described earlier [23]. The cells were plated on selective Brock Gelrite plates lacking uracil, which were supplemented with 0.2% dextrin and NZ amine. Colonies were grown at 75 °C during 6 days. The experiment was performed independently three times using quadruplicates of each strain.

## 3. Results

### 3.1. Nucleic Acid Binding Activity of SIRV2_Gp1

**Figure 1:**
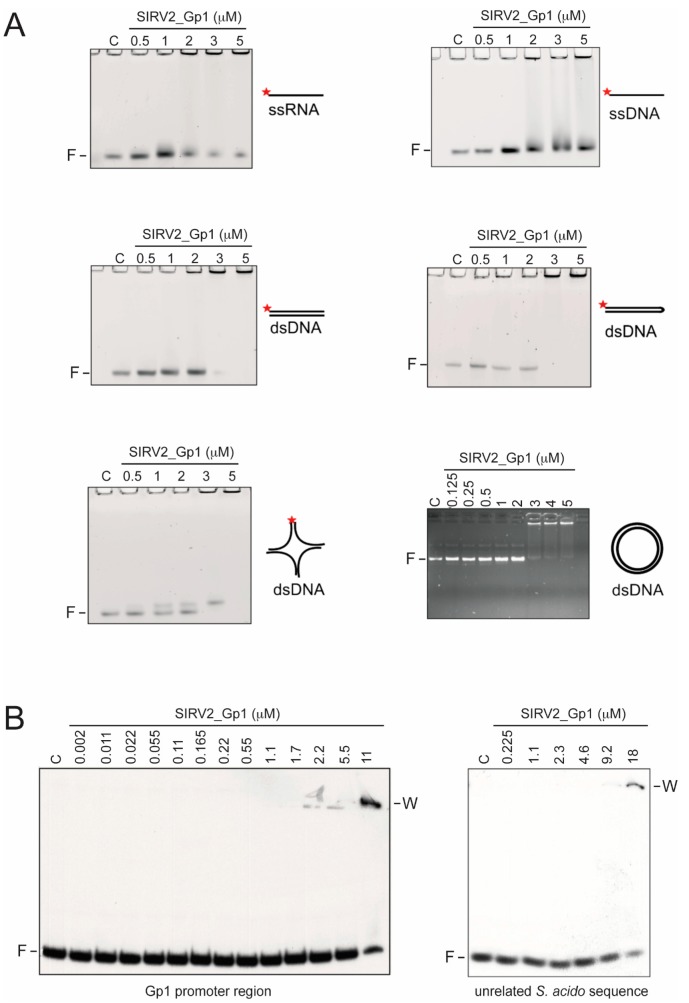
Nucleic-acid binding assays of SIRV2_Gp1 protein. (**A**) Fluorescence imaging of nucleo-protein adduct formation with SIRV2_Gp1 as seen after native gel electrophoresis of SIRV2_Gp1 with single-stranded (ss) RNA, ssDNA, double-stranded (ds) DNA, hairpin DNA and Holliday junction DNA, as indicated. The electrophoretic mobility shift assay (EMSA) experiment with plasmid DNA (right bottom panel) was not performed with fluorescently labeled DNA, but instead by ultraviolet (UV) imaging of an ethidium bromide-stained 1% agarose gel. Each substrate was incubated with different concentrations of the protein indicated in μM prior to being subjected to native gel electrophoresis. C indicates the control reaction without any protein. (**B**) EMSA of different concentrations of SIRV2_Gp1 with short ^32^P-labeled probes representing the Gp1 promoter sequence and an unrelated promoter sequence of *Sulfolobus acidocaldarius*, as indicated. Binding reactions were performed in presence of unlabeled competitor DNA. F, free probe. W, wells.

In order to assess whether or not SIRV2_Gp1 has a nucleic acid binding capacity, we heterologously expressed and purified SIRV2_Gp1 protein from *E. coli* by His-tag affinity and size exclusion chromatography. Induction of *SIRV2_gp1* expression inhibited growth of *E. coli* (data not shown). EMSAs were employed to analyze the interaction of this protein in vitro with a range of nucleic acids (Figure 1). Besides ssDNA and dsDNA probes, we also tested hairpin and Holliday junction DNA probes and an RNA probe, all with a randomized sequence. SIRV2_Gp1 displayed an interaction with all nucleic acid types, thereby causing the unbound probe to disappear (Figure 1A). Furthermore, a 2.7 kbp supercoiled plasmid DNA was tested for which a similar binding pattern was observed as for the short-randomized dsDNA probe. In all these binding experiments, higher-order nucleo-protein complexes were formed that were unable to penetrate the acrylamide or agarose gel during electrophoresis. Since the required protein concentrations in order to observe retardation exceeded 1 μM, these are low-affinity interactions. Also, the affinity and stability of the complexes are higher for ds than for ss nucleic acids, given the observed “smearing” and remaining unbound probe at the highest protein concentrations for ssRNA and ssDNA (Figure 1A).

To further analyze the sequence specificity of the observed SIRV2_Gp1-DNA interactions, we performed EMSA analysis using labeled DNA probes with a specific sequence in the presence of competing non-labeled random DNA (Figure 1B). We selected the control promoter region of the *SIRV2_gp1* gene as a putative specific target under the hypothesis that SIRV2_Gp1 is a specific transcription factor regulating its own expression, as is often the case for archaeal proteins with HTH domain. SIRV2_Gp1 only formed higher-order complexes upon the addition of relatively high protein concentrations (>10 μM), which were even higher than those required to shift the DNA in the assays with the random probes (Figure 1A). This can be explained by the absence of sequence specificity in the interaction, resulting in competition by the excess amounts of non-labeled competitor DNA added in the latter experiment. This is further confirmed by the observation of a similar binding behaviour for a probe with an irrelevant *S. acidocaldarius* sequence (Figure 1B). In conclusion, SIRV2_Gp1 displays nucleic acid binding activity and shows the highest affinity for dsDNA. Our data suggest that this DNA binding occurs without sequence specificity.

To verify whether or not SIRV2_Gp1 has a role in DNA transaction processes such as replication, we tested if SIRV2_Gp1 displays nuclease activity in addition to the binding activity, by incubating the protein with short fluorescently labeled DNA and RNA probes (see Materials and Methods) at 50 °C and separating the nucleic acid products on denaturing urea acrylamide gel (Figure S1). No cleaved oligonucleotide products were detected on the gel, suggesting that SIRV2_Gp1 does not have nuclease activity.

### 3.2. Atomic Force Microscopy Imaging of SIRV2_Gp1-DNA Complexes

**Figure 2:**
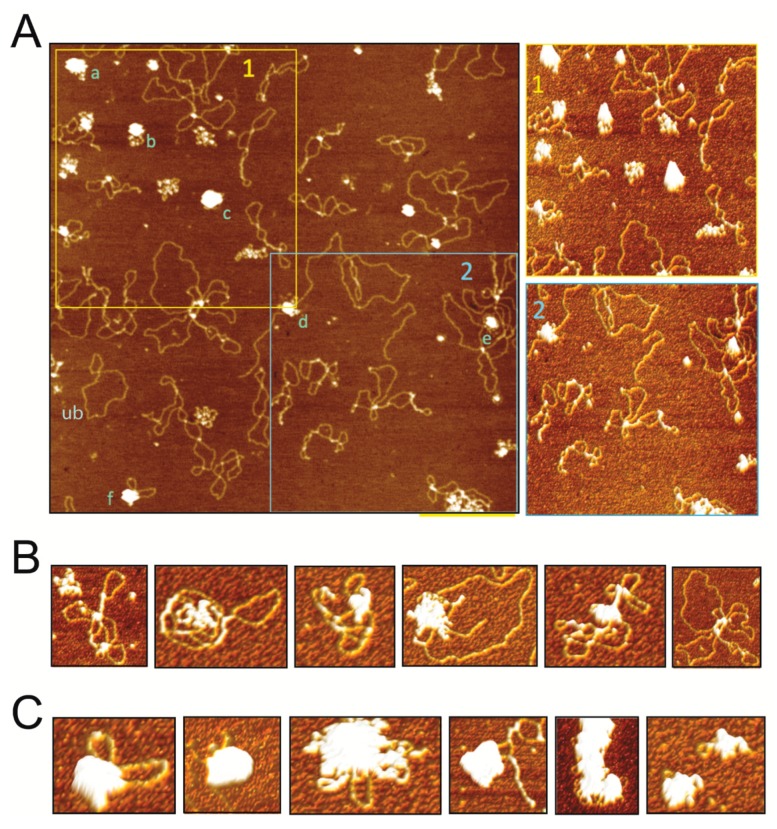
Atomic Force Microscopy (AFM) imaging of SIRV2_Gp1-DNA complexes. (**A**) A representative two-dimensional topographic AFM height image displaying unbound, small and strongly condensed complexes. The large complexes were characterized by a height of 3 to 6 nm: the vertical dimension of complex (a) is 5.2 nm, (b) is 4.2 nm, (c) is 6.2 nm, (d) is 3.1 nm, (e) is 3.1 nm, and (f) is 5.4 nm (see also Figure S2). Three-dimensional height images of area 1 (top right) and 2 (bottom right). A typical example of a presumably unbound DNA molecule is indicated with “ub”. (**B**,**C**) A selection of three-dimensional AFM images zoomed into a single complex, subdivided into classes 1 (**B**) and 2 (**C**), as explained in the text.

The observation of the interaction of SIRV2_Gp1 with plasmid DNA molecules (Figure 1) prompted us to further investigate the architecture of the formed nucleoprotein complexes by employing AFM imaging of single molecules (Figure 2). Within the same image, a heterogeneous population of SIRV2_Gp1-DNA complexes was observed, ranging from apparently relaxed plasmid DNA molecules, similarly as observed upon imaging a DNA-only sample (data not shown) and without clearly observable protein binding, to strongly condensed complexes harboring significant protein aggregation zones (Figure 2A). The co-occurrence of these populations reflects the highly cooperative nature of the interaction and supports the observation of a sudden transition from unbound DNA to higher-order protein-DNA molecules unable to penetrate the gel in the corresponding EMSA analysis (Figure 1A).

A qualitative analysis of AFM images that were recorded upon incubating plasmid DNA with 30 nM SIRV2_Gp1 demonstrated that the most commonly observed complexes (that also existed side-by-side) could be classified as one of two types (Figure 2B,C). While class 1 complexes (Figure 2B) are characterized by a single or few strongly aggregated regions besides a significant fraction of uncomplexed DNA, class 2 complexes (Figure 2C) are highly condensed protein-DNA aggregates in which almost the entire 2.7 kbp-sized DNA molecule is contained and small loops of DNA are occasionally still pointing outwards. The latter observation underscores that these are nucleoprotein complexes rather than aggregates solely composed of protein. It is clear that the binding by the small SIRV2_Gp1 protein causes a strong condensation of the DNA, forming large aggregates with approximate vertical dimensions between 3 and 6 nm (Figure 2A and Figure S2).

### 3.3. In Vivo Toxicity of SIRV2_Gp1

**Figure 3:**
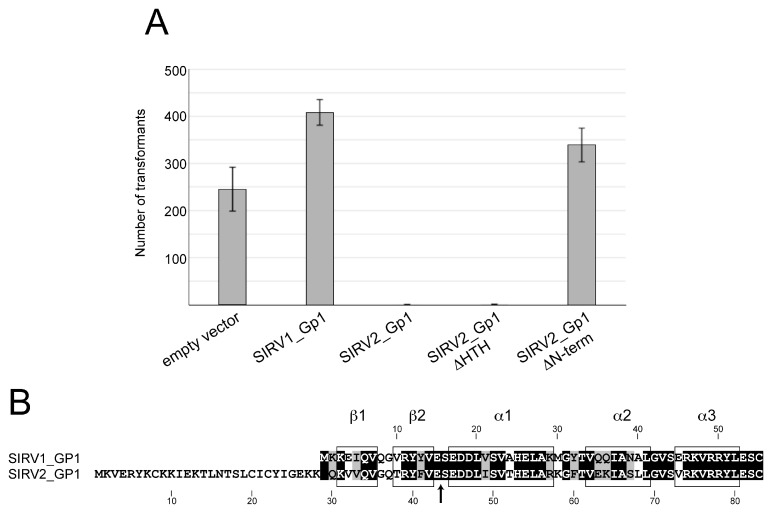
Toxicity effects of different Gp1 variants. (**A**) Transformation efficiencies of plasmid vectors harboring *SIRV*_*gp1* variants. Y-axis, number of transformants. An empty plasmid vector and plasmids containing *SIRV1*_*gp1*, *SIRV2*_*gp1* and truncations thereof were transformed into *S. acidocaldarius* and plated on selective medium. Colonies were counted after six days of incubation at 75 °C. The average absolute number of colonies is shown in the *y*-axis. Gp1 ΔHTH, Gp1 truncation mutant missing the helix-turn-helix (HTH) domain. Gp1 ΔN-term, Gp1 truncation mutant lacking the 28 N-terminal amino acids. Error bars, standard deviation. (**B**) Amino acid sequence alignment of SIRV2_Gp1 and SIRV1_Gp1, with indication of the secondary structure elements of the SIRV1_Gp1 structure. Arrow indicates C-terminus of Gp1 ΔHTH.

Upon observing the DNA binding and dramatic DNA condensation activity of SIRV2_Gp1, we hypothesized that this viral protein might influence the host cell viability. In the absence of a genetic system for Rudiviruses, we decided to study the influence of *SIRV2*_*gp1* expression using *S. acidocaldarius* that is a close relative of the host *S. islandicus* LAL14/1, and for which a genetic system is available [24]. The *SIRV2_gp1* gene was cloned in a maltose-inducible expression vector and transformed into *S. acidocaldarius* MW001. After six days, the number of transformants was counted (Figure 3A). While an empty control plasmid was transformed with high efficiency, those containing *SIRV2_gp1* did not yield any colonies. Since the maltose promoter is not very tightly regulated and leaky expression may occur without induction, these results suggest that the *SIRV2*_*gp1* product is toxic to the host cells.

We aimed to establish which part of the SIRV2_Gp1 protein is responsible for this toxic effect. BlastP analysis demonstrated that SIRV2_Gp1 shows highest sequence identity and similarity with SIRV1_Gp1 (Figure 3B). The main difference between both proteins is that the *SIRV1*_*gp1* ORF is predicted to encode a protein product lacking 28 amino acids at the N-terminus in comparison with the product of *SIRV2_gp1* (Figure 3B and Figure S3). Since the sequencing of the SIRV1 genome in this region was not complete [8], there are several unresolved base pairs just upstream of the annotated *SIRV1_gp1*, complicating complete annotation of this gene. We studied the importance of the 28 N-terminal amino acids and the HTH domain, respectively, for the toxic effects of SIRV2_Gp1 by expressing SIRV2_Gp1 truncation mutants in *S. acidocaldarius* (Figure 3A). Truncation mutants of the long SIRV2_Gp1 were either lacking the HTH domain (SIRV2_Gp1 ΔHTH), or the 28 N-terminal amino acids (SIRV2_Gp1 ΔN-term) (Figure 3). SIRV2_Gp1 ΔHTH exerted a toxic effect on *Sulfolobus* cells, as was the case for the wild type SIRV2_Gp1 protein, as almost no transformants were observed. In contrast, SIRV1_Gp1 yielded a similar number of transformants as the control empty plasmid (~200–400). Also in the case of SIRV2_Gp1 ΔN-term transformation efficiencies were comparable to transformation with the empty control plasmid. Hence, it can be concluded that the N-terminal domain of SIRV2_Gp1 is responsible for an extreme reduction in viability when expressed in *Sulfolobus* cells.

### 3.4. DNA-Binding Characteristics of SIRV1_Gp1 and a Truncated SIRV2_Gp1 Variant

**Figure 4:**
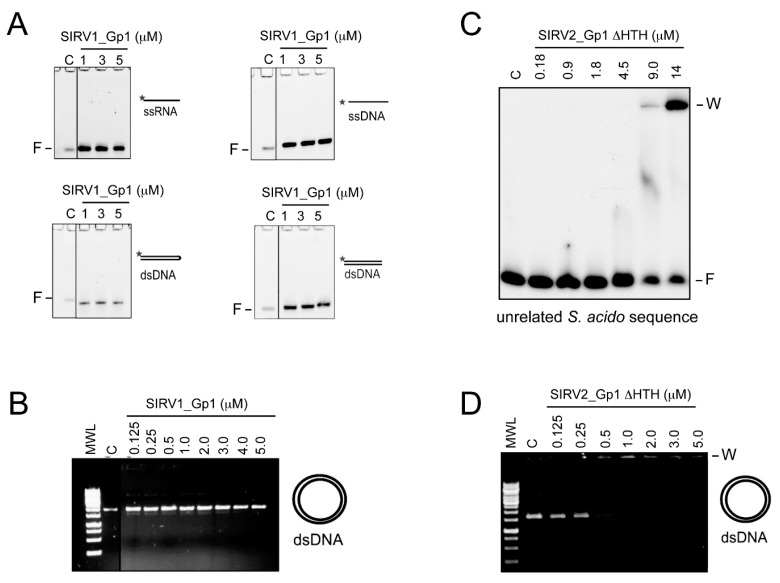
Nucleic-acid binding assays of SIRV1_Gp1 and SIRV2_Gp1 ∆HTH proteins. (**A**) Fluorescence imaging of nucleo-protein adduct formation with SIRV1_Gp1 with short randomized probes. C indicates the control reaction without any protein. (**B**) EMSA of SIRV1_Gp1 with plasmid DNA visualized by UV imaging of an ethidium bromide-stained 1% agarose gel. (**C**) EMSA of SIRV2_Gp1 ∆HTH with plasmid DNA visualized by UV imaging of an ethidium bromide-stained 1% agarose gel. (**D**) EMSA of SIRV2_Gp1 ∆HTH with a short ^32^P-labeled probe representing an unrelated promoter sequence of *Sulfolobus acidocaldarius*. Binding reactions were performed in presence of unlabeled competitor DNA. F, free probe; W, wells.

To assess whether or not the toxicity mediated by the 28 amino-acid N-terminus of SIRV2 was linked with the DNA-binding and -condensation characteristics, DNA-binding behavior of the shorter SIRV1_Gp1 protein was analyzed (Figure 4A). This demonstrated that, in the same binding reaction conditions as applied for SIRV2_Gp1, SIRV1_Gp1 does not bind nucleic acids. This is a surprising observation because the HTH motif is present in both homologs with an almost identical recognition helix α3 (7 out of 8 α3 residues are conserved (Figure 3B)), which is typically directly involved in DNA binding.

The involvement of the N-terminal domain of SIRV2_Gp1 in DNA binding was further investigated by subjecting a recombinantly purified preparation of the SIRV2_Gp1 ∆HTH truncation variant to DNA-binding analysis. Similarly, as upon heterologously overexpressing the full-length SIRV2_Gp1 protein in *E. coli*, growth of the cells was hampered during the expression of SIRV2_Gp1 ∆HTH (data not shown). This observation is in agreement with the toxicity observed in *S. acidocaldarius* (Figure 3A). EMSAs demonstrated that SIRV2_Gp1 ∆HTH interacted with both supercoiled plasmid DNA as with a short DNA probe (Figure 4B). The observed binding behaviour is the same as observed for the full-length protein, with the formation of higher-order nucleoprotein complexes that hardly penetrate the gel. Shifting of the DNA, whether circular plasmid DNA or short linear DNA fragments, occurs at somewhat lower protein concentrations for SIRV2_Gp1 ∆HTH than for the full-length protein, suggesting that the truncated protein displays a higher affinity. We can thus conclude that the N-terminal domain of SIRV2_Gp1 mediates DNA interactions while the HTH motif in Gp1 proteins does not display any DNA-binding activity. The SIRV2_Gp1 ∆HTH mutant is 45 amino acids long and composed of the 28-amino acid extension specific of SIRV2_Gp1 and, additionally, the two β strands β 1 and β 2. We hypothesize that it is the lysine-rich N-terminal stretch that is responsible for the observed DNA binding and not β 1 and β 2, since the latter are also present in the SIRV1_Gp1 homolog, which displays a high sequence identity with SIRV2_Gp1 (Figure 3B).

### 3.5. Structure of SIRV1_Gp1

**Figure 5:**
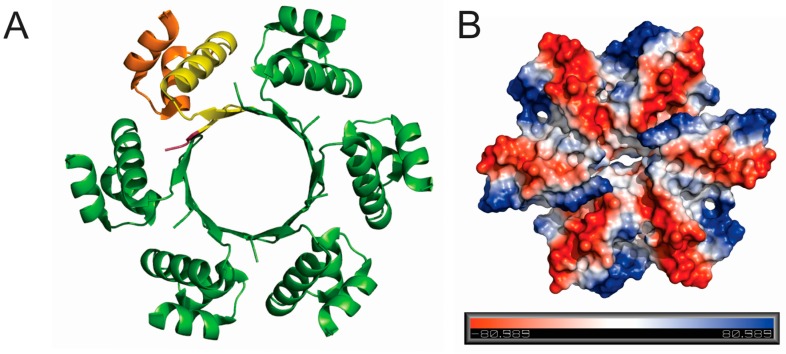
Crystal structure of SIRV1_Gp1. (**A**) Hexameric conformation in which the protein was crystallized. One individual subunit is depicted in yellow. The HTH domain is highlighted in orange and the N-terminus is shown in purple. (**B**) Surface representation showing electrostatic potential.

The interesting DNA-interaction abilities of SIRV2_Gp1 led us to further study its structure. A structure of the short SIRV1_Gp1 purified from *E. coli* is available (PDB ID: 2X48 [20]) and displays, besides the C-terminal HTH motif, two β strands that mediate oligomerization, thereby assembling the protein into a hexameric ring-like structure with the HTH motifs pointing outwards (Figure 3B and Figure 5A). While one side of the ring carries generally no charge, the other side displays alternating positive and negative charged areas, stretching from the outside to the inner cavity of the ring (Figure 5B). Based on this structure, we performed homology modeling of SIRV2_Gp1, using PHYRE2 software [25]. The C-terminal part of SIRV2_Gp1, containing the HTH domain, was modeled with high fidelity on the SIRV1_Gp1 structure, whereas the 28 N-terminal amino acids of SIRV2_Gp1 could not be modeled. The N-terminus of SIRV1_Gp1 is located on the inner side of the ring formed when in hexameric conformation. Thus, it is likely that the N-terminus of SIRV2_Gp1 is pointing outwards perpendicular to the ring. ITASSER software [26] predicted (confidence score of ~70%) that the N-terminus of SIRV2_Gp1 might be partly in alpha-helical conformation. Based on our observations described above, it can be concluded that this N-terminal stretch is responsible for the observed DNA interactions of SIRV2_Gp1 and that the outwards pointing HTH motifs do not display DNA-binding activity under the tested conditions.

## 4. Discussion

In this study, we demonstrated that the Rudiviral protein SIRV2_Gp1 binds several nucleic acid species with a preference for dsDNA. This binding appears to lack sequence specificity given the observation that SIRV2_Gp1 significantly retards migration of short randomized or large plasmid DNA probes (Figure 1 and Figure 4). However, we cannot exclude the possibility that SIRV2_Gp1 might bind a yet unidentified sequence with higher specificity. Furthermore, study of the architecture of SIRV2_Gp1 nucleoprotein complexes revealed protein-induced aggregation zones in dense complexes. Employing the *S. acidocaldarius* genetic system, we further showed that SIRV2_Gp1 is toxic to *Sulfolobus* cells and that this toxicity is caused by a lysine-rich N-terminal extension, which also mediates DNA binding and in which the typical HTH motif does not seem to be involved. The shorter SIRV1 version of Gp1 was not toxic to *Sulfolobus* cells and EMSAs indicated that this protein is unable to interact with DNA.

Upon aligning *SIRV1*_*gp1* and *SIRV2*_*gp1* DNA sequences (Figure S3), the correctness of ORF annotation could be questioned. To analyze the transcriptional structure of the *SIRV1*_*gp1* gene, we aimed at analyzing transcriptome data. While the many repeats encoded in this genome region have hampered a Northern blot expression analysis of *SIRV1*_*gp1* [27], the stable replication and high virus production of SIRV2 have allowed for a recent RNA-seq analysis [18]. In this study, transcription levels of *SIRV2*_*gp1* were quantified at several time points during infection. Based on these data, it appears that the *SIRV2*_*gp1* gene is characterized by a transcriptional dynamic resulting in two alternative transcripts that are translated from different start codons yielding the full-length and truncated SIRV2_Gp1 protein, respectively. At early stages of infection, hardly any reads covering the 5′-region of *SIRV2*_*gp1* were detected, suggesting that, at that time point, possibly only a short version of *gp1*, encoding the SIRV1_Gp1 homolog lacking the N-terminal extension, is expressed [18]. However, later during SIRV2 infection the long version of the *gp1* gene appears to be transcribed, although the coverage of the 5′-region is still considerably lower than the 3′-region [18]. Therefore, the shorter 55 amino-acid version of SIRV2_Gp1 might be the dominant species during SIRV2 infection, while at later stages the longer 83 amino-acid protein might become relevant. The massive DNA condensation caused by the 83 amino-acid version and its apparent toxicity might be compatible to a role in elimination of the host defense system. The absence of the longer Gp1 version in SIRV1, and the subsequent absence of DNA condensation, wrapping activity and toxicity, seems in concert with the observed mild and partially defective progression of infection by SIRV1.

The observation of the N-terminal extension of the full-length SIRV2_Gp1 protein mediating host toxicity by DNA condensation does not inform us about the putative function of the truncated version expressed during early stages of infection and of the corresponding SIRV1_Gp1 ortholog. Previously, it was shown that the SIRV2_Gp1 protein interacts with a Holliday junction resolvase (encoded by ORF121 in SIRV2) [18] and the PCNA3 (proliferating cell nuclear antigen) subunit of the *Sulfolobus* sliding clamp, a processivity factor of archaeal DNA polymerase [28]. Based on this observation, SIRV2_Gp1 was hypothesized to be implicated in the initiation of viral genome replication and/or the resolution of viral replicative intermediates [28,29]. It could thus be envisaged that SIRV2_Gp1 has a dual function, depending on its translational length, and that it assists in viral replication during early stages of the infection while condensing the host genome during later stages. The lack of observed nucleic acid-binding activity in vitro for SIRV1_Gp1, despite the presence of the HTH motif, was unexpected given the unequivocal implication of this motif in DNA binding. Possibly, the assembly into a hexameric ring in vitro (Figure 5) prevents interaction with DNA because of a suboptimal relative positioning with respect to consecutive helical turns of a DNA molecule. In vivo, a heterooligomeric assembly of SIRV1_Gp1 (or the truncated SIRV2_Gp1 protein) and the resolvase might harbour DNA-binding activity.

The massive DNA wrapping and condensation activity as observed for SIRV2_Gp1 might be employed as an inducible toxic peptide in a biotechnological setting for containment of the spread of genetically modified organisms or as a viral weapon for killing pathogenic bacteria. In addition to this biotechnological relevance, our findings contribute to the understanding of the Rudiviral infection cycle and pave the way for further study of archaeal viruses in general.

## Supplementary Materials

The following are available online at www.mdpi.com/1999-4915/9/7/190/s1, Figure S1: Cleavage assay of SIRV1_Gp1 and SIRV2_Gp1; Figure S2: Cross-section analysis of a selection of large complexes; Figure S3: Alignment of SIRV1_gp1 and SIRV2_gp1 on the base pair and amino acid level; Table S1: Sequences of oligonucleotides used in this work.
