# Supplementary material for: DNA-Interacting Characteristics of the Archaeal Rudiviral Protein SIRV2_Gp1
Source: Viruses. 2017 Jul 18;9(7):190. doi: 10.3390/v9070190 (PMC5537682; doi:10.3390/v9070190)
Supplement: Supplementary file 1 [file viruses-09-00190-s001.pdf]

Supplementary Material

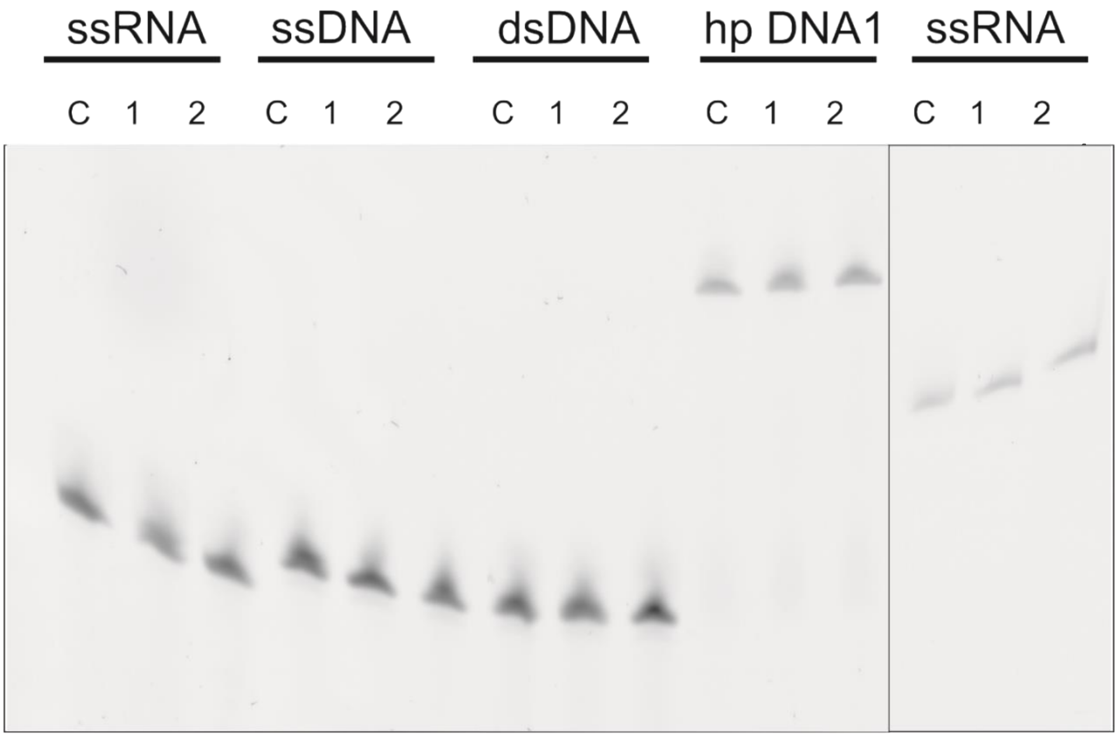

Figure S1.

Cleavage assay of SIRV1_Gp1 and SIRV2_Gp1. Fluorescence imaging of nucleic acid products after denaturing gel electrophoresis of reactions of both proteins with different types of fluorescently labeled nucleic acids. C, loading control without protein. 1, SIRV1_Gp1. 2, SIRV2_Gp1. hpDNA, hairpin DNA.

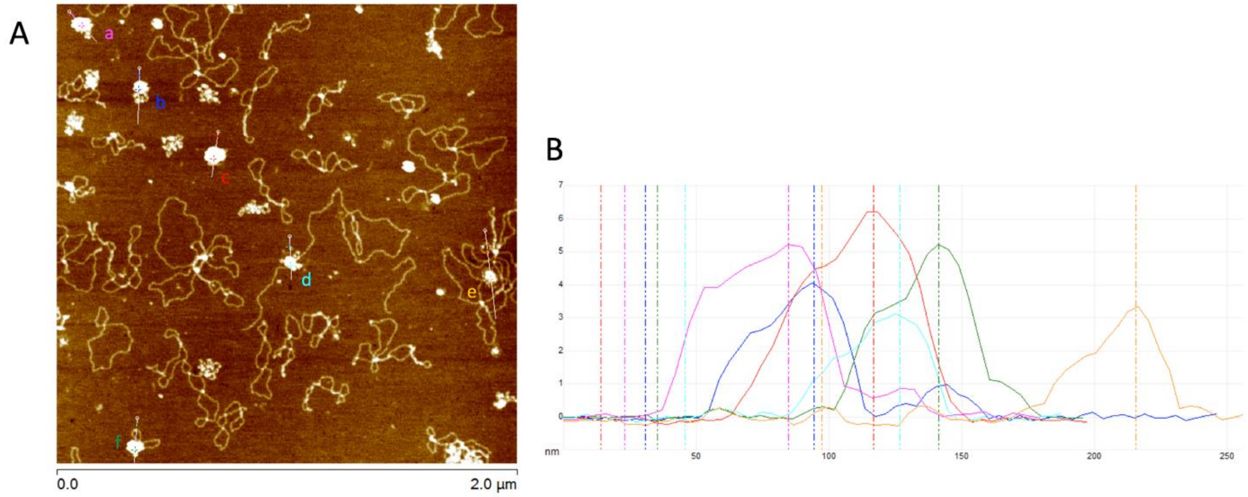

Figure S2.

Cross-section analysis of a selection of large complexes (Figure 2A). (A) Height image with indicated complexes (a-f). (B) Cross-sections of the indicated complexes.

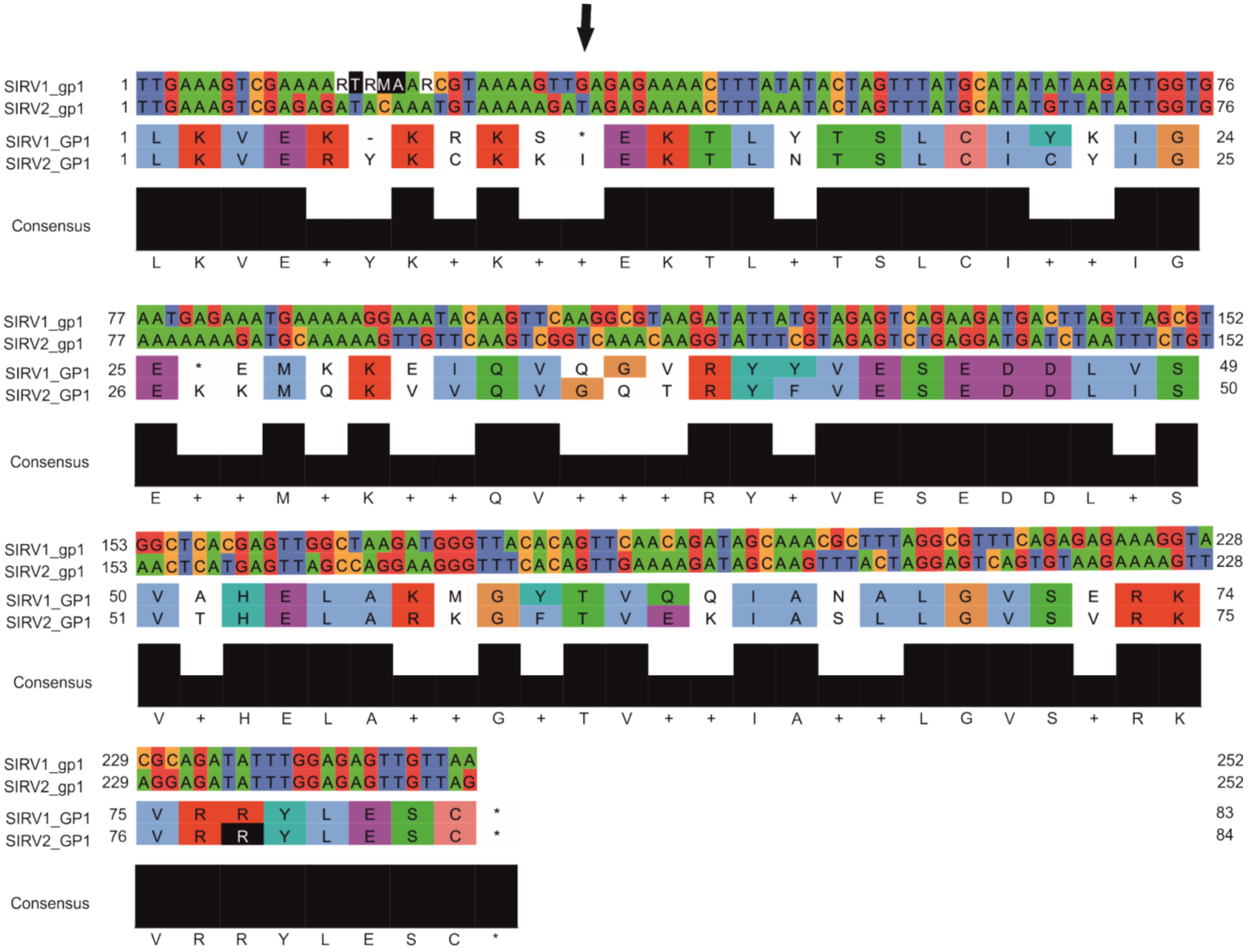

Figure S3.

Alignment of SIRV1_*gp1* and SIRV2_*gp1* on the base pair and amino acid level. Truncation of SIRV1_*gp1* in comparison with SIRV2_*gp1* indicated with arrow.

Table S1. Sequences of oligonucleotides used in this work

**Number/name**	**Sequence**
1	ATGAAAGTCGAGAGATACAAATG
2	ACAACTCTCCAAATATCTCCTAAC
3	TGAGTTTTTTTCATTTTTTGCGTAAATTTC
4	GAAATTTACGCAAAAAATGAAAAAAACTCA
5	TGAGTTTTTTTCATTTTTTGCGTAAATTTCGAAATTTACGCAAAAAATG AAAAAAACTCA
6	AGTAAGTTTTTCTTTGGTTTTTTCTCATTA
7	GCGTTACAATGGAAACTATTCTTGGCAGTTGCATCCAACG
8	CGTTGGATGCAACTGCCAAGAATAGTGTCAGTTCCAGACG
9	CGTCTGGAACTGACACTATTCTTGGCGAATGGTCGTAAGC
10	GCTTACGACCATTCGCCAAGAATAGTTTCCATTGTAACGC
11	AAATACTAGTTTATGCATATGTTATATTGGTGAAAAAAAGATGAAAA AGGAAATACAAGTTC
12	ACAACTCTCCAAATATCTGCGTACCTTTCTCTCTGAAAC

13	TTGAAAGTCGAGAGATACAAATGTAAAAAGATAGAGAAAACTTTAA ATACTAGTTTATGCATATG
14	GGAGATACCCTTATGAAAGTCGAGAG
15	CTCTCGACTTTCATAAGGGTATCTCC
16	CACCATGAAAAAGGAAATACAAGTTCAAGG
17	TTAACAACTCTCCAAATATCTG
18	CACCATGCAAAAAGTTGTTCAAGTCGGTC
19	CTAACAACTCTCCAAATATCTCCTA
20	CACCATGAAAGTCGAGAGATACAAATG
21	TTACTCAGACTCTACGAAATAC
22	CTCAGACTCTACGAAATACC
ep399	TAGCTAACTAGCTAGATAGTAG
ep400	ACATTTGTATCTCTCGACTTTC
ep092	ATACCCTGTCTGTTCTCTTC
ep093	CAATACTCATTTTAATCTCGCC
LL139	TCCACACCGTGGTTCACTAA
LL140	GATGGGAGTCTTGGACTCTAGG
